# Regulating interface Schottky barriers toward a high-performance self-powered imaging photodetector†

**DOI:** 10.1039/d2ra04820e

**Published:** 2022-09-12

**Authors:** Jun Yan, Feng Gao, Weiqiang Gong, Yongzhi Tian, Lin Li

**Affiliations:** Key Laboratory for Photonic and Electronic Bandgap Materials, Ministry of Education, School of Physics and Electronic Engineering, Harbin Normal University Harbin 150025 China physics_lin@hotmail.com lil@hrbnu.edu.cn; School of Physics and Engineering, Zhengzhou University Zhengzhou 450001 China

## Abstract

Two-dimensional (2D) layered organic–inorganic hybrid perovskites have attracted wide attention in high-performance optoelectronic applications due to their good stability and excellent optoelectronic properties. Here, a high-performance self-powered photodetector is realized based on an asymmetrical metal–semiconductor–metal (MSM) device structure (Pt-(PEA)_2_PbI_4_ SC-Ag), which introduces a strong built-in electric field by regulating interface Schottky barriers. Benefitting from excellent built-in electrical potential, the photodetector shows attractive photovoltaic properties without any power supply, including high photo-responsivity (114.07 mA W^−1^), fast response time (1.2 μs/582 μs) and high detectivity (4.56 × 10^12^ Jones). Furthermore, it exhibits high-fidelity imaging capability at zero bias voltage. In addition, the photodetectors show excellent stability by maintaining 99.4% of the initial responsivity in air after 84 days. This work enables a significant advance in perovskite SC photodetectors for developing stable and high-performance devices.

## Abstract 2

A facile method of regulating interface Schottky barriers is used for self-powered photodetectors. Benefitting from the excellent built-in electrical potential, the detector can exhibit high-fidelity imaging capability.

## Graphical abstract

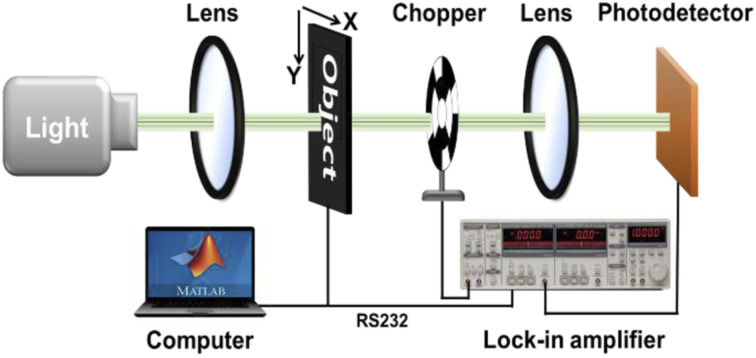

## Introduction

Photodetectors can convert incident photons into electrical signals. As the key building block of state-of-the-art optoelectronic products, photodetectors have widespread application in the field of environmental surveillance, optical communication, biological and image sensing, *etc.*^1–9^ From the application point of view, photodetectors with high responsivity, energy saving, fast response speed, and stable operation are very important.^10,11^ However, traditional photodetectors have to rely on an external power source to complete photodetection,^12–14^ which not only increases the cost but also increases the complexity of the system. If a photodetector works in a self-powered manner (namely self-power photodetector), it can work at an external voltage of 0 V, which has the characteristics of small size, low weight, easy integration, *etc.*,^15–17^ and it can largely reduce energy consumption, which is beneficial to society, especially in the severe situation of increasing global energy demand and extreme environmental applications. Hence, the self-powered photodetector has received increasing attention recently. Until now, the self-powered photodetectors have realized by using the photovoltaic effect by constructing Schottky junction, *pn* junction, and heterojunction structures.^18–21^ Among these device structures, metal–semiconductor–metal (MSM) based structures have attracted much attention due to their simple fabrication process, compatibility with large-area imaging devices, and ease of integration.^18,22^ The asymmetric electrode regulation is an effective method to fabricate Schottky junctions based on MSM structure, which can form a built-in electric field due to the two metal electrodes with different work functions.^4,23–26^ Thereby, the Schottky junction can effectively separate the photogenerated carriers without applying an external bias.

The semiconductor materials with strong light absorption, high carrier mobility and simple fabrication process are very crucial for obtaining high-performance photodetectors. Currently, most commercially available photodetectors rely on traditional semiconductors such as indium gallium arsenide (InGaAs), silicon (Si), *etc.*, which are typically fabricated by using high-temperature, complicated, and expensive high-vacuum deposition processes.^27–31^ In recent years, organic–inorganic hybrid perovskites have been widely used in many optoelectronic devices such as solar cells, light-emitting diodes and photodetectors, *etc.*^32–37^ As one of the most promising semiconductor optoelectronic materials, perovskites possess a variety of attractive physical and optoelectronic properties, such as strong light absorption coefficient, high carrier mobility, long carrier diffusion length, solution-based processability, *etc.*^38–45^ Recently, the layered two-dimensional (2D) halide perovskite has a general formula (RNH_3_)_2_PbX_4_, where R is a long-chain alkyl or aromatic group, and X is a halogen.^46,47^ In layered 2D perovskites, the inorganic lead halide octahedra (PbX_6_) are separated by organic cations, and the stacking layer is piled up along the vertical direction under the van der Waals forces. The 2D layered perovskites tend to self-organize into the quantum well structures, in which the photocarriers are mainly confined in inorganic layers, carrier transport is anisotropic, and interlayer transport is limited.^48–50^ Hence, 2D perovskites are more suitable for planar structures, which are beneficial to the collection of photogenerated carriers. Zhang *et al.* fabricated perovskite 2D (PEA)_2_PbBr_4_ single crystal (SC) and assembled with a planar photodetector structure of Au/(PEA)_2_PbBr_4_/Au, the photodetector shows broad spectral response and excellent optoelectronic properties.^51^ Liu's group realized a broadband photodetection using 2D (PEA)_2_PbI_4_ perovskite SC-based photodetectors. By changing the crystal plane of the SC, the planar structure photodetector fabricated in the (001) plane exhibits more excellent performance.^52^ In addition, 2D perovskites possess large organic cations, which are more hydrophobic than smaller organic cations such as MA^+^, FA^+^ and thus exhibit excellent moisture and photostability. Therefore, designing and fabricating 2D perovskite SC self-powered planar photodetector structure is crucial for improving device performance.

In this work, we presented a high-performance and stable perovskite SC self-powered photodetector based on Pt-(PEA)_2_PbI_4_-Ag (Pt-Ag) MSM structure. The asymmetric metal electrodes with different work functions introduce a built-in electric field by adjusting the interfacial Schottky barrier, which makes the device have photovoltaic properties and realize self-powered photodetection. The detector shows a wide spectral response range from ultraviolet to visible light and exhibits high performance without any external power supply, including high photo-responsivity (114.07 mA W^−1^), a fast response time (1.2 μs/582 μs), high detectivity (4.56 × 10^12^ Jones). Moreover, the photodetectors without encapsulation exhibit high stability in air environments. During the 12 week measurement, the device maintained good photodetection capability, which maintains 99.4% of the initial responsivity, and after continuous illumination operation, which exhibited negligible deviation behavior. In addition, high-fidelity image was obtained using the Pt-(PEA)_2_PbI_4_-Ag photodetector as the sensing pixel of the imaging system. The above results indicate that the Pt-(PEA)_2_PbI_4_-Ag photodetector exhibits great potential in future high-sensitivity self-powered optoelectronic applications.

## Results and discussion

**Fig. 1:**
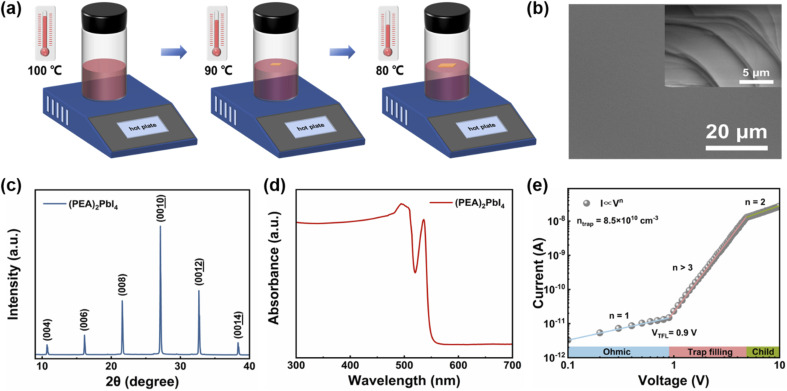
(a) Schematic illustration of the crystallization process. (b) The (001) plane and (010) plane (inset) SEM images of (PEA)_2_PbI_4_ SC. (c) and (d) The XRD and the absorption spectra of (PEA)_2_PbI_4_ SC, respectively. (e) The *I*–*V* characteristic of (PEA)_2_PbI_4_ SC in the dark investigated by the space charge limited current method.

The schematic illustration of the preparation for (PEA)_2_PbI_4_ SC is presented in Fig. 1a. 2D (PEA)_2_PbI_4_ SCs were grown by a controlled cooling crystallization process (detailed experiment procedures in Experimental section). Fig. S1 (ESI†) shows the photographs of the (PEA)_2_PbI_4_ SCs completed at different temperatures during the cooling crystallization process. The scanning electron microscope (SEM) image of (PEA)_2_PbI_4_ SC is shown in Fig. 1b. The SEM image of the (001) plane shows a very smooth surface, while the image of the (010) plane (inset) shows a periodic layered morphology, fully consistent with the (PEA)_2_PbI_4_ SC layered structure. This is because the basic building blocks of these compounds consist of an inorganic layer of PbI_6_ octahedra, with a layer of PEA^+^ cations covering both sides of the lead halide layers through hydrogen bonds between ammonium groups and iodine atoms, and adjacent layers are interconnected by weak van der Waals forces.^49,51^ Hence, a periodic layered morphology is displayed in Fig. 1b. The X-ray diffraction (XRD) spectra were used to investigate the crystal structure of the (PEA)_2_PbI_4_ SC. Fig. 1c shows the XRD pattern of the (PEA)_2_PbI_4_ SC and the well-defined diffraction peaks are corresponding to the (00*h*) series of reflections, indicating its good crystallinity and the high degree of preferred orientation. Fig. 1d shows the optical absorption spectrum of (PEA)_2_PbI_4_ SC. The absorption peak at 536 nm (2.31 eV) is caused by excitons confined in the PbI_4_ quantum well.^52^ The absorption spectrum reveals an exciton binding energy of 2.31 eV and a continuum absorption spectrum at 527 nm (2.35 eV). As shown in Fig. S2 (ESI†), the PL peak of (PEA)_2_PbI_4_ SC is at 536 nm, which demonstrates that exciton absorption defines the absorption onset. The absorption spectra show band-edge cutoffs and no absorption tails, indicating that the perovskite SC prepared by the simple cooling crystallization process has low defect states. To investigate the trap density (*n*_trap_) of the (PEA)_2_PbI_4_ SC, the dark *I–V* characteristic of the device was determined using the space charge limited current (SCLC) method. As shown in Fig. 1e, as the voltage increased, the *I* ∝ *V*^*n*^ curves exhibit three different regions including an ohmic region (*n* = 1), trap filling region (*n* > 3) and Child's (*n* = 2) region. The trap-filled voltage (*V*_TFL_) was used to calculate the trap density (*n*_trap_), which can be calculated with the following equation:1
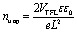
where *V*_TFL_ is the trap-filled limit voltage, *ε* is the relative dielectric constant for (PEA)_2_PbI_4_ SC, *ε*_0_ is the vacuum permittivity, *e* is the elementary charge, and *L* is the thickness of the (PEA)_2_PbI_4_ SC. *ε* is obtained by measurement the capacitance between two electrodes, as detailed in Fig. S3 (ESI†) and Note S1 (ESI†). As shown in Fig. 1e, the *n*_trap_ is measured as low as 8.5 × 10^10^ cm^−3^.

**Fig. 2:**
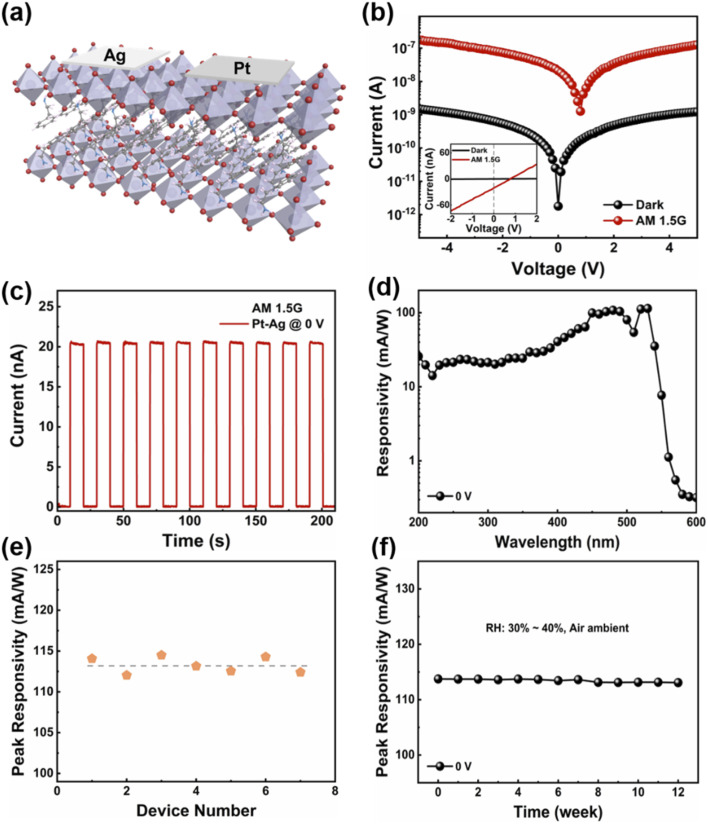
(a) Schematic illustration of Pt–Ag photodetector. (b) The *I–V* curves for the Pt–Ag photodetector in dark and under a solar simulator (AM 1.5G, 100 mW cm^−2^). Inset shows the linear *I–V* curves of the photodetector under dark and illumination. (c) The time-dependent current of the Pt–Ag photodetector under periodically switched light illumination (AM 1.5G, 100 mW cm^−2^) at 0 V. (d) The photoresponse curve of the Pt–Ag photodetector at 0 V. (e) The peak responsivity of seven Pt–Ag photodetectors in a batch. (f) The spectral responsivity stability of Pt–Ag photodetector at 0 V as a function of time.

By using an asymmetric electrode pair to regulate interface Schottky barriers, a built-in electric field is generated, and the separation of photogenerated carriers can be achieved without applying any bias voltage in the photodetector. To verify the applicability of asymmetric electrodes, a Pt-(PEA)_2_PbI_4_-Ag (Pt-Ag) photodetector was constructed using Pt and Ag as electrodes. Fig. 2a shows a schematic diagram of the device structure of the photodetector, and the SEM image of the photodetector is shown in Fig. S4 (ESI†). Fig. 2b shows the *I–V* curves under dark and illumination (AM 1.5G, 100 mW cm^−2^). The *I–V* curves of the Pt–Ag devices were measured in the voltage range from −5 V to 5 V, and it was observed clearly that the device showed a low dark current. Obviously, the *I–V* curve deviates from the origin of the coordinates when the photodetector is under illumination, resulting in an obvious photovoltaic effect, the current increased from 1.78 × 10^−12^ A to 2.08 × 10^−8^ A at 0 V, and a high *I*_light_/*I*_dark_ of 1.16 × 10^4^. Therefore, the Pt–Ag photodetector can work without an external power supply. In addition, the control devices with Pt-(PEA)_2_PbI_4_-Pt (Pt–Pt) and Ag-(PEA)_2_PbI_4_-Ag (Ag-Ag) symmetric electrodes were fabricated to further illustrate the photovoltaic effect of asymmetric electrode devices. Fig. S5 (ESI†) shows the linear *I–V* curves of the symmetric electrode device under dark and illumination (AM 1.5G, 100 mW cm^−2^). It can be observed that under illumination, the *I–V* curves all pass through the coordinate origin, which further proves that devices with symmetrical electrodes do not have photovoltaic effects. The photocurrent of the device with a symmetric electrode is smaller than that of the asymmetric electrode device under illumination at 5 V bias. It is shown that the photodetector with asymmetric electrodes can not only achieve the capability of self-powered detection but also improve the optoelectronic performance of the device.

Furthermore, the time-dependent current (*I*–*t*) curve of the Pt–Ag photodetector is investigated in self-powered mode when the light illumination is periodically switched on the photodetector (Fig. 2c). As the illumination is turned on and off, the photogenerated current increases rapidly and stabilizes, and then decreases rapidly, indicating that the Pt–Ag device has good on/off switching characteristics. In addition, the photocurrent did not change significantly during ten repeated cycles, indicating its good stability and repeatability. As an important factor in determining the sensitivity of the detector, the response efficiency to optical signals is evaluated by the spectral responsivity, and it can be expressed by the following formula:^53,54^2
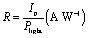
where *I*_p_ is the photocurrent and *P*_light_ is the incident light power. Fig. 2d shows the responsivity spectrum of the planar structure Pt–Ag photodetector at 0 V. The detector exhibits an obvious broad spectral response in the wavelength range from 200 nm to 570 nm, which is consistent with the absorption spectrum of (PEA)_2_PbI_4_ SC (Fig. 1d). The peak responsivity is 114.07 mA W^−1^ at 530 nm. The detectivity (D*) is also a key parameter in defining the performances of self-powered photodetectors. The D* reflects the detection capability of the photodetector to weak light which can be calculated with the following equation:^10,55,56^3
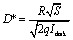
where *R* is the responsivity of the photodetector, *S* is the effective area, and *I*_dark_ is the dark current. Therefore, the D* values of the self-powered photodetector are calculated and shown in Fig. S6a (ESI†). The D* value is as high as 4.56 × 10^12^ Jones at 530 nm, suggesting a great potential for detecting weak light. The EQE (external quantum efficiency) spectrum of the Pt–Ag photodetector was recorded at 0 V (Fig. S6b, ESI†), *R* is a function of EQE, which is calculated by the following formula:^57^4
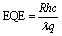
where *h*, *c*, and *q* are Planck constant, the velocity of light, and the unit charge, respectively. The EQE value for the photoresponse is 26.74% at 530 nm. Fig. S6c (ESI†) shows the responsivity spectrum of the vertical structure Pt–Ag photodetector at 0 V. Obviously, the responsivity of the planar structure is much higher than that of the vertical structure, which is attributed to the poor carrier transport efficiency caused by the interlayer confinement of 2D perovskites. The photoresponse spectra of the Pt–Ag photodetector with different bias voltages are shown in Fig. S7a (ESI†), the responsivity at 530 nm increases from 183.69 mA W^−1^ at 1 V to 714.93 mA W^−1^ at 5 V. Obviously, the peak response increases linearly with the increase of applied bias voltage because of the direct dependence of the extraction efficiency of photogenerated carriers with bias voltages. To demonstrate the advantage of the asymmetric electrode structure, Fig. S7b and c (ESI†) show the responsivity spectra of symmetric electrode photodetectors with different bias voltages. The responsivity spectral with symmetric Pt electrodes is shown in Fig. S7b (ESI†). The curves still show a distinctly broad spectral response, with the responsivity at 530 nm increasing from 103.77 mA W^−1^ at 1 V to 389.36 mA W^−1^ at 5 V. Fig. S7c (ESI†) shows the responsivity spectral of the symmetric Ag electrodes device. The responsivity wavelength range is also from 200 nm to 570 nm and the responsivity at 530 nm increased from 129.88 mA W^−1^ at 1 V to 468.53 mA W^−1^ at 5 V. It is worth noting that the responsivity of devices with symmetric electrodes is lower than that of the device with asymmetric electrodes. Because of the synergy effect of the built-in electric field and external electric field, increases the separation and collection efficiency of photo-generated electrons and hole in the detection region.

To investigate the reproducibility of asymmetric electrode devices, the responsivity of seven individually prepared Pt–Ag photodetectors was measured under 0 V bias. As shown in Fig. 2e, the as-prepared photodetector exhibited good reproducibility with an average value of 113.28 mA W^−1^ for the peak responsivity. Self-powered photodetectors usually work under extreme conditions for a long time, so stability is also one of the important parameters for evaluating device performance. As shown in Fig. 2f, the peak responsivity of the Pt–Ag device after being exposed to the air environment for 12 weeks, which maintains 99.4% of the initial responsivity. To further prove the operational stability of the self-powered device, the *I*–*t* curves of an asymmetric electrode Pt–Ag photodetector under periodic switching illumination for 3000 s at 0 V was investigated (Fig. S8, ESI†). By comparing the last five cycles with the first five cycles, the device has negligible deviation over the test period, indicating a reliable light operation operational stability of the device.

**Fig. 3:**
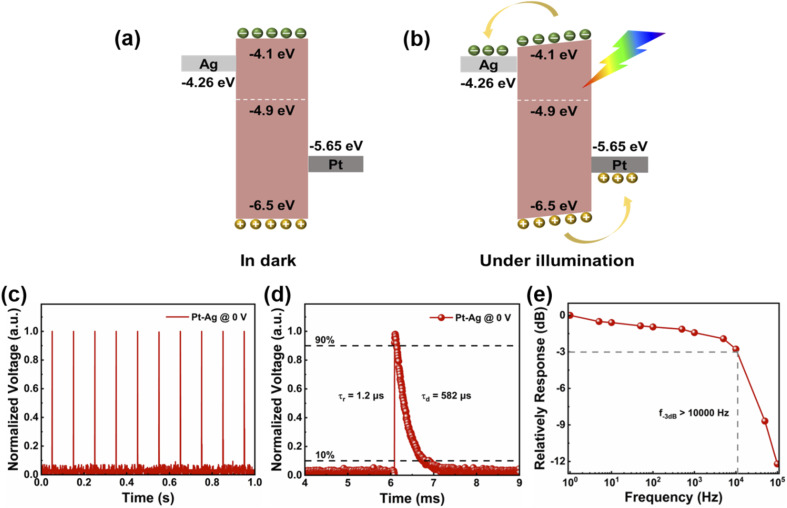
(a) and (b) The energy band diagrams of the device with Pt–Ag asymmetric electrodes in dark and under illumination. (c) and (d) Time-resolved response of the Pt–Ag photodetector at 0 V. (e) The frequency response of Pt–Ag photodetector.

In order to explain the working mechanism of the self-powered photodetector with asymmetric electrodes, it can be illustrated by analyzing the energy band diagram and electron transfer process of the device. Due to the different work functions between the SC and the metal electrode, charge transfer occurs when the two materials are in contact with each other, thereby forming a Schottky barrier at the SC–metal contact interface. The built-in electric field generated in the device channel is composed of two opposing is determined by the Schottky barrier height difference between the metal contacts.^58^ For devices with symmetrical electrodes (Ag–Ag, Pt–Pt), the Schottky barrier distribution is mirror-symmetric (Fig. S9, ESI†). The photogenerated carriers have no preferred drift direction within the device when the photon energy irradiated on the device surface is higher than the (PEA)_2_PbI_4_ SC band gap.^50^ The photocurrents generated near the two metal electrodes are opposite and symmetrical, resulting in zero net photocurrents.^59,60^ Therefore, a bias voltage needs to be applied to separate photogenerated carriers and generate photocurrent. As shown in Fig. 3a, due to the different work functions of the two metal electrodes, different Schottky barrier heights will be generated, and the mirror symmetry in the Schottky barrier distribution is broken (Fig. 3b), thus creating a built-in electric field in the device channel, which generates photocurrent without any bias applied. Specifically, in the Pt–Ag device, electrons will flow from the (PEA)_2_PbI_4_ SC (Ag) to Pt ((PEA)_2_PbI_4_ SC) due to the lower (higher) work function of Pt (Ag) relative to the Fermi level of the SC. Furthermore, a built-in electric field from the (PEA)_2_PbI_4_ SC (Ag) to the Pt ((PEA)_2_PbI_4_ SC) direction is formed near the metal electrode contact, and the built-in electric field direction of the metal electrodes on both sides is the same and is superimposed on the active layer to introduce a higher built-in electric field. Therefore, the generated photogenerated carriers are separated by the built-in electric field and transferred to the corresponding metal electrodes without bias voltage, leading to generation of the photocurrent at 0 V bias.

The response speed of the photodetector is another important parameter to evaluate the photoresponse characteristics of the photodetector. It determines the ability to track rapidly changing light signals. Under the excitation of 355 nm pulsed laser (pulse width: 5 ns, laser power: 2 mW), the transient photoresponse of the Pt–Ag photodetector was measured at 0 V (Fig. 3c and d). The photoresponse time is defined as the time required to increase from 10% to 90% of the maximum photocurrent (rise time, *τ*_r_) and decrease from 90% to 10% of the maximum photocurrent (decay time, *τ*_d_).^61,62^ As shown in Fig. 3c, the photoresponse speed of the Pt–Ag photodetector was very fast, stable, and reproducible. The *τ*_r_ and *τ*_d_ of the photodetector are estimated to be 1.2 μs and 582 μs (Fig. 3d). At the same time, the response speed of Pt–Pt and Ag–Ag was measured at 1 V (Fig. S10, ESI†). It also shows fast, repeatable, and stable response (Fig. S10a and c, ESI†). The Pt–Pt device *τ*_r_ and *τ*_d_ were measured to be 7.3 μs and 606 μs, respectively (Fig. S10b, ESI†). The *τ*_r_ and *τ*_d_ of the Ag–Ag device are 4.5 μs and 620 μs, respectively (Fig. S10d, ESI†). The Pt–Ag photodetector exhibits faster response speed, indicating that the photodetectors can track fast-changing optical signals. The characteristic parameters of this self-powered photodetector with asymmetric electrodes are in the same order of magnitude or even better than self-powered photodetectors reported in recent years (Table 1). To further characterize the photoelectric performance, the normalized response of the Pt–Ag device at different frequencies is recorded in Fig. 3e, showing a fast photoresponse with a −3 dB cutoff frequency greater than 10 000 Hz. It demonstrates that the device has a stable response to high frequency signals.

**Table 1:** The comparison of key performance parameters of self-powered photodetectors with asymmetric electrodes

Materials	Responsivity (mA W^−1^)	Detectivity (Jones)	*τ*_r_/*τ*_d_	Ref.
Au/MAPbI_3_ MWs/Ag	160	1.3 × 10^12^	13.8/16.1 μs	21
FTO/MAPbBr_3_/MAPbI_*x*_Br_3−*x*_/Au	11.5	—	2.3/2.76 s	63
ITO/CsPbBr_3_:ZnO/Ag	11.5	—	409/17.92 ms	64
ITO/BaTiO_3_/Ag	3 × 10^−4^	3 × 10^5^	400/400 ms	65
Au/GaAs/Ti	6.45	1.4 × 10^11^	—	66
Pt/MAPbBr_3_/Au	2	1.4 × 10^10^	70/150 μs	67
FTO/TiO_2_/MAPbBr_3_/Bi_2_Te_3_	97.5	1.66 × 10^12^	28/32 ms	68
Pt/(PEA)_2_PbI_4_/Ag	114.07	4.56 × 10^12^	1.2/582 μs	This work

**Fig. 4:**
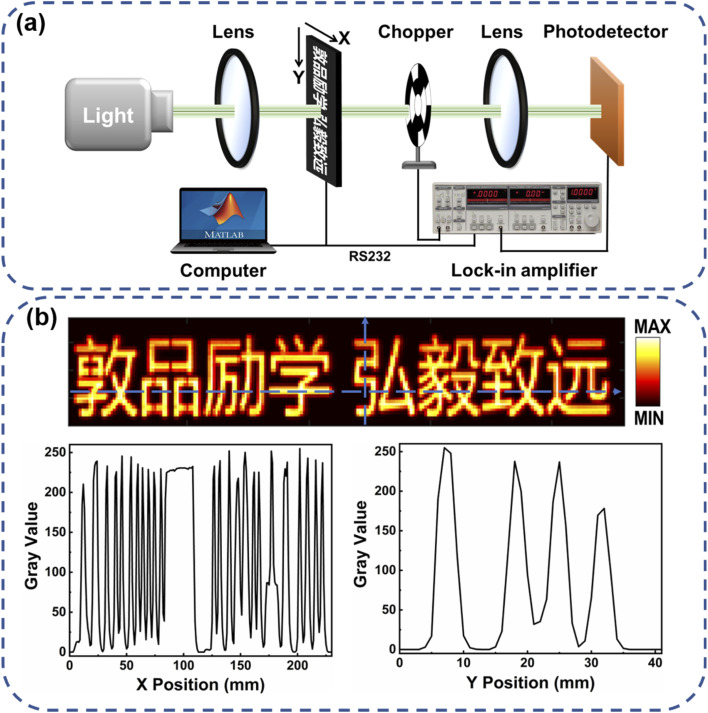
(a) Schematic illustration of the imaging system employing the Pt–Ag photodetector as sensing pixel. (b) Image result obtained from the imaging system, and gray value along the line marked in the image.

To investigate the imaging ability of the self-powered Pt–Ag photodetector at 0 V, an imaging system was constructed employing the photodetector as a sensing pixel, as shown in Fig. 4a. The object for imaging systems with hollow patterns was made on steel foils with a laser cutter. The object is mounted on a computer-controlled translation stage that can move continuously in both horizontal and vertical directions. Using a 490 nm laser (3 mW) as the light source, with the movement of the translation stage, the photocurrent is extracted and recorded by the lock-in amplifier and computer, and the position coordinates of the obtained object are recorded simultaneously. Then, a 41 × 230 pixels image was obtained by converting the output signal current to a “Gray code” (Fig. 4b). The images show clear boundaries that are highly consistent with the shape of the target object (Fig. S11, ESI†). These results demonstrate the Pt–Ag photodetector can satisfy the requirement of an imaging system in self-powered mode.

## Conclusions

In summary, we have prepared 2D perovskite SC by cooling crystallization process, and a self-powered photodetector has been developed by designing asymmetric electrodes on (PEA)_2_PbI_4_ SC. The asymmetric metal electrodes with different work functions introduce a built-in electric field that endows the device with photovoltaic characteristics, which enables the device to realize self-powered detection. The photodetector exhibits excellent performance for detection of photo signals without any external power supply, including high photo-responsivity (114.07 mA W^−1^), fast response time (1.2 μs/582 μs), high detectivity (4.56 × 10^12^ Jones), proving its great potential for practical applications. Moreover, the photodetectors without encapsulation exhibit high stability in the air, with little change in peak responsivity after 12 weeks, which maintains 99.4% of the initial responsivity, and after continuous illumination operation, which exhibited negligible deviation behavior. In addition, high-fidelity images were obtained using the Pt–Ag photodetector as the sensing pixel of the imaging system. Overall, the self-powered photodetectors with asymmetric electrodes provide enormous potential in research on fundamental physics and practical applications.

## Experimental section

### Chemicals

PbI_2_ (99.999%) and C_6_H_5_C_2_H_4_NH_3_I (PEAI) were purchased from Xi'an Polymer Light Technology Corp. γ-butyrolactone (GBL, ≥99%) was purchased from Sigma-Aldrich. All chemicals were used as received without further purification.

### Preparation of (PEA)_2_PbI_4_ single crystal (SC)

PbI_2_ and PEAI (1 : 2 molar ratio) were dissolved in GBL. The solution was stirred at 100 °C overnight to form a clear solution (2 M). Then, the solution was cooled to 90 °C at a cooling rate of 0.5 °C h^−1^, and small crystals were formed on the surface of the solution. As the temperature continued to decrease to 80 °C, the small crystals became larger but still floated on the surface of the solution. Then, a (PEA)_2_PbI_4_ SC with a size of 3–5 mm was obtained.

## Photodetector fabrication

To fabricate metal–semiconductor–metal (MSM) photodetectors, contact electrodes were deposited on perovskite SCs by vacuum thermal evaporation using a shadow mask. The device channel length of 100 μm and a width of 1 mm. In order to construct a photodetector with asymmetric contact electrodes, a high work function metal electrode (50 nm Pt) was deposited on the one side of perovskite SC. Then, the position of the evaporated electrode was covered, and a low work function metal electrode (50 nm Ag) was deposited on the surface of the single crystal by repeating the above process. The effective illuminated area of the device is about 1 × 0.1 mm^2^.

### Characterization

The morphology of the sample was obtained by using a Hitachi SU-70 scanning electron microscope (SEM). The crystalline property of the SC was analyzed by X-ray diffractometer (XRD). The optical absorption spectra were recorded using an ultraviolet-visible spectrophotometer. The photoresponse characteristic (Zolix DR800-CUST) of the PD was measured at RT. *I–V* and *I*–*t* curves were measured by using a Keithley 2400 source meter and a xenon-lamp-based solar simulator. The self-powered photodetector used for stability measurement was stored in a conventional environment in the air. The response time for the device was measured at 0 and 1 V by using a Tektronix TDS3032C digital phosphor oscilloscope with pulsed laser excitation at 355 nm. All the detectors were characterized without encapsulation in the ambient atmosphere at room temperature.

## Author contributions

L. L. conceived the project. J. Y. wrote the draft of the manuscript. J. Y. and Y. T. designed and synthesized the PDs. J. Y., F. G., and W. G. characterized the performance of the PDs. Y. T. characterized the single-pixel imaging capability of the PDs. The manuscript was written through contributions of all authors. All authors have given approval for the final version of the manuscript.

## Conflicts of interest

There are no conflicts to declare.

## Supplementary Material

RA-012-D2RA04820E-s001

## References

[cit1] Li C., Wang H., Wang F., Li T., Xu M., Wang H., Wang Z., Zhan X., Hu W., Shen L. (2020). Light: Sci. Appl..

[cit2] Li L., Chen H., Fang Z., Meng X., Zuo C., Lv M., Tian Y., Fang Y., Xiao Z., Shan C., Xiao Z., Jin Z., Shen G., Shen L., Ding L. (2020). Adv. Mater..

[cit3] Rauch T., Böberl M., Tedde S. F., Fürst J., Kovalenko M. V., Hesser G., Lemmer U., Heiss W., Hayden O. (2009). Nat. Photonics.

[cit4] Hao D., Liu D., Zhang S., Li L., Yang B., Huang J. (2022). Adv. Opt. Mater..

[cit5] Konstantatos G., Howard I., Fischer A., Hoogland S., Clifford J., Klem E., Levina L., Sargent E. H. (2006). Nature.

[cit6] Li C. L., Lu J. R., Zhao Y., Sun L. Y., Wang G. X., Ma Y., Zhang S. M., Zhou J. R., Shen L., Huang W. (2019). Small.

[cit7] Zhu H., Fu Y., Meng F., Wu X., Gong Z., Ding Q., Gustafsson M. V., Trinh M. T., Jin S., Zhu X. Y. (2015). Nat. Mater..

[cit8] Zhou H., Chen Q., Li G., Luo S., Song T.-B., Duan H.-S., Hong Z., You J., Liu Y., Yang Y. (2014). Science.

[cit9] Li S. X., Xu X. L., Yang Y., Xu Y. S., Xu Y., Xia H. (2021). ACS Appl. Mater. Interfaces.

[cit10] Hao D., Liu D., Shen Y., Shi Q., Huang J. (2021). Adv. Funct. Mater..

[cit11] Jiang D. L., Li L., Chen H. Y., Gao H., Qiao Q., Xu Z. K., Jiao S. J. (2015). Appl. Phys. Lett..

[cit12] Leung S. F., Ho K. T., Kung P. K., Hsiao V. K. S., Alshareef H. N., Wang Z. L., He J.-H. (2018). Adv. Mater..

[cit13] Lu H., Tian W., Cao F., Ma Y., Gu B., Li L. (2016). Adv. Funct. Mater..

[cit14] Zhou J. C., Chu Y. L., Huang J. (2016). ACS Appl. Mater. Interfaces.

[cit15] Chen H., Liu K., Chen X., Zhang Z., Fan M., Jiang M., Xie X., Zhao H., Shen D. (2014). J. Mater. Chem. C.

[cit16] Fang H., Zheng C., Wu L., Li Y., Cai J., Hu M., Fang X., Ma R., Wang Q., Wang H. (2019). Adv. Funct. Mater..

[cit17] Cao F., Meng L., Wang M., Tian W., Li L. (2019). Adv. Mater..

[cit18] Veeramalai C. P., Yang S., Zhi R., Sulaman M., Saleem M. I., Cui Y., Tang Y., Jiang Y., Tang L., Zou B., Perumal Veeramalai C., Yang S., Zhi R., Sulaman M., Saleem M. I., Cui Y., Tang Y., Jiang Y., Tang L., Zou B. (2020). Adv. Opt. Mater..

[cit19] Konstantatos G., Sargent E. H. (2010). Nat. Nanotechnol..

[cit20] Wang Y., Wu T., Barbaud J., Kong W., Cui D., Chen H., Yang X., Han L. (2019). Science.

[cit21] Wu C. Y., Peng W., Fang T., Wang B., Xie C., Wang L., Yang W. H., Luo L. B. (2019). Adv. Electron. Mater..

[cit22] Ito M., Kumai T., Hamaguchi H., Makiuchi M., Nakai K., Wada O., Sakurai T. (1985). Appl. Phys. Lett..

[cit23] Gao W., Zhang S., Zhang F., Wen P., Zhang L., Sun Y., Chen H., Zheng Z., Yang M., Luo D., Li H. (2020). Adv. Electron. Mater..

[cit24] Shaikh P. A., Shi D., Retamal J. R. D., Sheikh A. D., Haque M. A., Kang C.-F., He J.-H., Bakr O. M., Wu T. (2016). J. Mater. Chem. C.

[cit25] Saidaminov M. I., Haque M. A., Almutlaq J., Sarmah S., Miao X.-H., Begum R., Zhumekenov A. A., Dursun I., Cho N., Murali B. (2017). Adv. Opt. Mater..

[cit26] Maculan G., Sheikh A. D., Abdelhady A. L., Saidaminov M. I., Haque M. A., Murali B., Alarousu E., Mohammed O. F., Wu T., Bakr O. M. (2015). J. Phys. Chem. Lett..

[cit27] Yao M., Jiang J., Xin D., Ma Y., Wei W., Zheng X., Shen L. (2021). Nano Lett..

[cit28] Ma N., Jiang J., Zhao Y., He L., Ma Y., Wang H., Zhang L., Shan C., Shen L., Hu W. (2021). Nano Energy.

[cit29] Zhao Y., Li C., Jiang J., Wang B., Shen L. (2020). Small.

[cit30] Liu Z., Liu X., Sun B., Tan X., Ye H., Zhou J., Tang Z., Shi T., Liao G. (2020). Adv. Mater. Technol..

[cit31] Yang J., Pi M., Zhang D., Tang X., Du J. (2021). Chin. J. Lumin..

[cit32] Min H., Lee D. Y., Kim J., Kim G., Lee K. S., Kim J., Paik M. J., Kim Y. K., Kim K. S., Kim M. G., Shin T. J., Seok S. I. (2021). Nature.

[cit33] Gong W., Tian Y., Yan J., Gao F., Li L. (2022). J. Mater. Chem. C.

[cit34] Lei Y., Chen Y., Zhang R., Li Y., Yan Q., Lee S., Yu Y., Tsai H., Choi W., Wang K., Luo Y., Gu Y., Zheng X., Wang C., Wang C., Hu H., Li Y., Qi B., Lin M., Zhang Z., Dayeh S. A., Pharr M., Fenning D. P., Lo Y., Luo J., Yang K., Yoo J., Nie W., Xu S. (2020). Nature.

[cit35] Yang L., Ma X., Zheng S., Chen C., Dai Q., Song H. (2020). Chin. J. Lumin..

[cit36] Li S. X., Xu Y. S., Li C. L., Guo Q., Wang G., Xia H., Fang H. H., Shen L., Sun H. B. (2020). Adv. Mater..

[cit37] Palabathuni M., Akhil S., Singh R., Mishra N. (2022). ACS Appl. Nano Mater..

[cit38] Dutt V. G. V., Akhil S., Mishra N. (2020). ChemNanoMat.

[cit39] Jeon N. J., Noh J. H., Yang W. S., Kim Y. C., Ryu S., Seo J., Seok S. I. (2015). Nature.

[cit40] Zhao Y., Zhu K. (2016). Chem. Soc. Rev..

[cit41] Sum T. C., Mathews N. (2014). Energy Environ. Sci..

[cit42] García de Arquer F. P., Armin A., Meredith P., Sargent E. H. (2017). Nat. Rev. Mater..

[cit43] Filip M. R., Eperon G. E., Snaith H. J., Giustino F. (2014). Nat. Commun..

[cit44] Li S. X., Xia H., Sun X. C., An Y., Zhu H., Sun H. B. (2022). Adv. Funct. Mater..

[cit45] Akhil S., Dutt V. G. V., Mishra N. (2021). ChemNanoMat.

[cit46] Zhang S., Audebert P., Wei Y., Al Choueiry A., Lanty G., Bréhier A., Galmiche L., Clavier G., Boissière C., Lauret J.-S., Deleporte E. (2010). Materials.

[cit47] Di J., Chang J., Liu S. (2020). EcoMat.

[cit48] Xiao X., Dai J., Fang Y., Zhao J., Zheng X., Tang S., Rudd P. N., Zeng X. C., Huang J. (2018). ACS Energy Lett..

[cit49] Lin Y., Bai Y., Fang Y., Wang Q., Deng Y., Huang J. (2017). ACS Energy Lett..

[cit50] Li H., Song J., Pan W., Xu D., Zhu W. A., Wei H., Yang B. (2020). Adv. Mater..

[cit51] Xiao X., Dai J., Fang Y., Zhao J., Zheng X., Tang S., Rudd P. N., Zeng X. C., Huang J. (2018). ACS Energy Lett..

[cit52] Liu Y., Ye H., Zhang Y., Zhao K., Yang Z., Yuan Y., Wu H., Zhao G., Yang Z., Tang J., Xu Z., Liu S. (2019). Matter.

[cit53] Zhou H., Song Z., Grice C. R., Chen C., Zhang J., Zhu Y., Liu R., Wang H., Yan Y. (2018). Nano Energy.

[cit54] Wu D., Zhou H., Song Z., Zheng M., Liu R., Pan X., Wan H., Zhang J., Wang H., Li X., Zeng H. (2020). ACS Nano.

[cit55] Chen X., Wei Z. (2021). Chin. J. Lumin..

[cit56] Yu P., Hu K., Chen H., Zheng L., Fang X. (2017). Adv. Funct. Mater..

[cit57] Wu D., Xu Y., Zhou H., Feng X., Zhang J., Pan X., Gao Z., Wang R., Ma G., Tao L., Wang H., Duan J., Wan H., Zhang J., Shen L., Wang H., Zhai T. (2022). InfoMat.

[cit58] Yu W. J., Liu Y., Zhou H., Yin A., Li Z., Huang Y., Duan X. (2013). Nat. Nanotechnol..

[cit59] Britnell L., Ribeiro R. M., Eckmann A., Jalil R., Belle B. D., Mishchenko A., Kim Y. J., Gorbachev R. V., Georgiou T., Morozov S. V., Grigorenko A. N., Geim A. K., Casiraghi C., Castro Neto A. H., Novoselov K. S. (2013). Science.

[cit60] Liu Y., Cheng R., Liao L., Zhou H., Bai J., Liu G., Liu L., Huang Y., Duan X. (2011). Nat. Commun..

[cit61] Pan X., Zhang J., Zhou H., Liu R., Wu D., Wang R., Shen L., Tao L., Zhang J., Wang H. (2021). Nano-Micro Lett..

[cit62] Shuang Z., Zhou H., Wu D., Zhang X., Xiao B., Ma G., Zhang J., Wang H. (2022). Chem. Eng. J..

[cit63] Cao M., Tian J., Cai Z., Peng L., Yang L., Wei D. (2016). Appl. Phys. Lett..

[cit64] Li C., Han C., Zhang Y., Zang Z., Wang M., Tang X., Du J. (2017). Sol. Energy Mater. Sol. Cells.

[cit65] Ma N., Zhang K., Yang Y. (2017). Adv. Mater..

[cit66] Nusir A. I., Manasreh M. O. (2015). IEEE Electron Device Lett..

[cit67] Shaikh P. A., Shi D., Retamal J. R. D., Sheikh A. D., Haque M. A., Kang C.-F., He J.-H., Bakr O. M., Wu T. (2016). J. Mater. Chem. C.

[cit68] Liu S., Jiao S., Lu H., Yang S., Zhao Y., Wang D., Gao S., Wang J., Zhao L., Li Y. (2022). Adv. Opt. Mater..

