# Supplementary material for: Regulating interface Schottky barriers toward a high-performance self-powered imaging photodetector
Source: RSC Adv. 2022 Sep 12;12(40):25881–9. doi: 10.1039/d2ra04820e (PMC9465635; doi:10.1039/d2ra04820e)
Supplement: RA-012-D2RA04820E-s001 [file RA-012-D2RA04820E-s001.pdf]

Electronic Supplementary Material (ESI) for RSC Advances.
This journal is © The Royal Society of Chemistry 2022

Supporting information

Regulating interface Schottky barriers toward high-performance self-powered imaging photodetector

Jun Yan[a], Feng Gao[a], Weiqiang Gong[a], Yongzhi Tian[b] and Lin Li[a,*]

[a] Key Laboratory for Photonic and Electronic Bandgap Materials, Ministry of Education, School of Physics and Electronic Engineering, Harbin Normal University
Harbin 150025, China.
E-mail: physics_lin@hotmail.com; lil@hrbnu.edu.cn
[b] School of Physics and Engineering, Zhengzhou University, Zhengzhou, 450001, China.

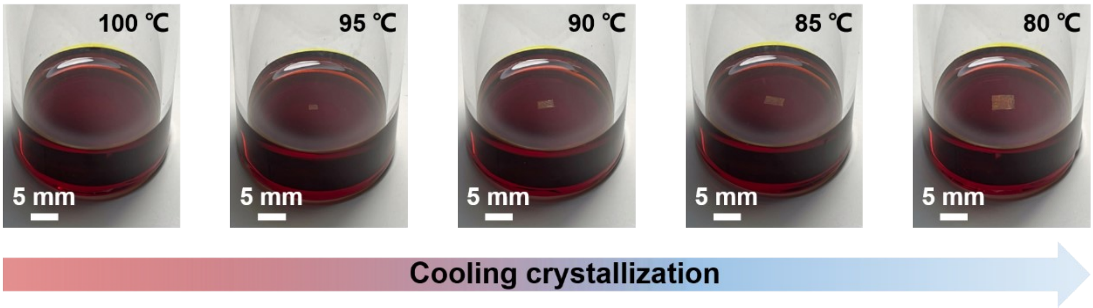

Fig. S1 The photograph of the $(PEA)_2PbI_4$ SCs grown with decreasing temperature.

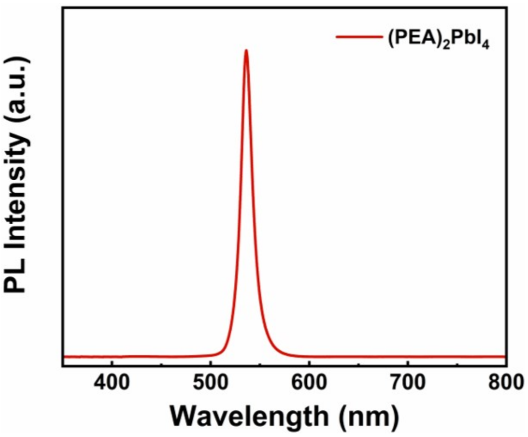

Fig. S2 The PL spectra of (001) plane of the $(PEA)_2PbI_4$ SC.

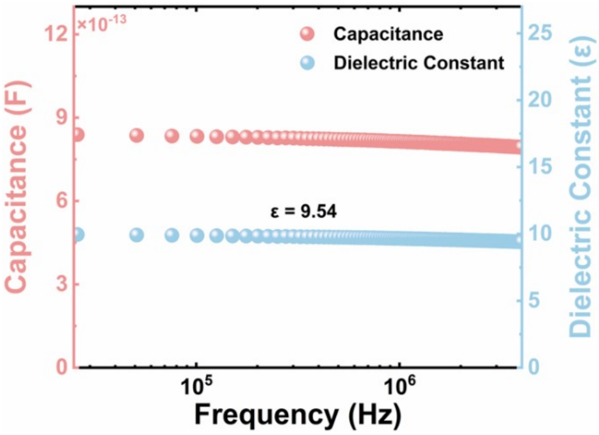

Fig. S3 Frequency-dependent capacitance and dielectric constant curves for the $(PEA)_2PbI_4$ SC device.

Note S1. The calculation of the relative dielectric constant of the $(PEA)_2PbI_4$ SC.

A simple plate capacitor was built to measure the relative dielectric constant of $(PEA)_2PbI_4$ SC. Device configuration was Au/$(PEA)_2PbI_4$ SC/Au where the $(PEA)_2PbI_4$ SC was served as the dielectric layer. By measuring the capacitance-frequency at dark, as shown in Fig. S2, the relative dielectric constant of the $(PEA)_2PbI_4$ SC was obtained by using the parallel plate capacitor model:

$$C = \frac{\varepsilon\varepsilon_0 S}{d}$$

where S is the electrode area, d is the thickness of SCs, ε_0 is the vacuum dielectric constant.

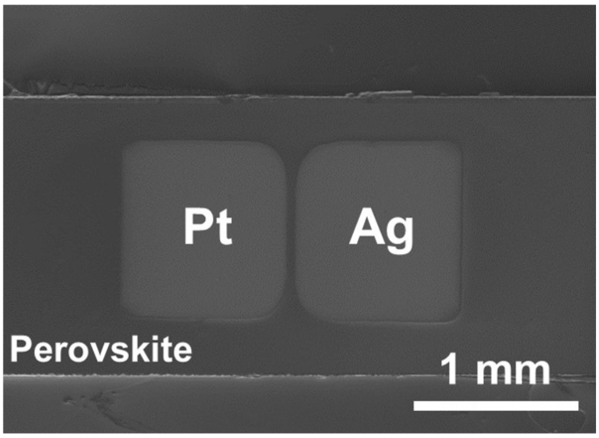

Fig. S4 The SEM image of the Pt-$(PEA)_2PbI_4$-Ag device.

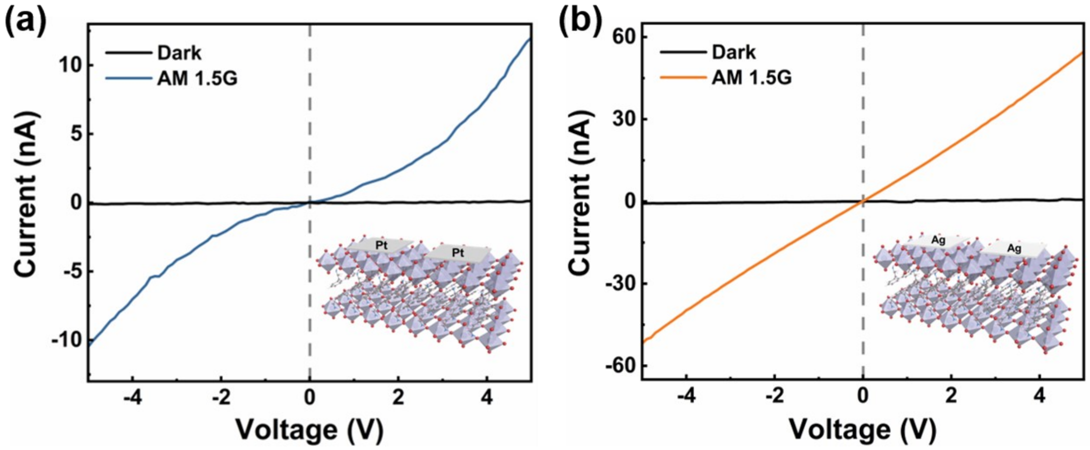

Fig. S5 The linear *I–V* curves for the photodetector in dark and under a solar simulator (AM 1.5G, 100 mW cm^{-2}), (a) Pt-Pt device and (b) Ag-Ag device.

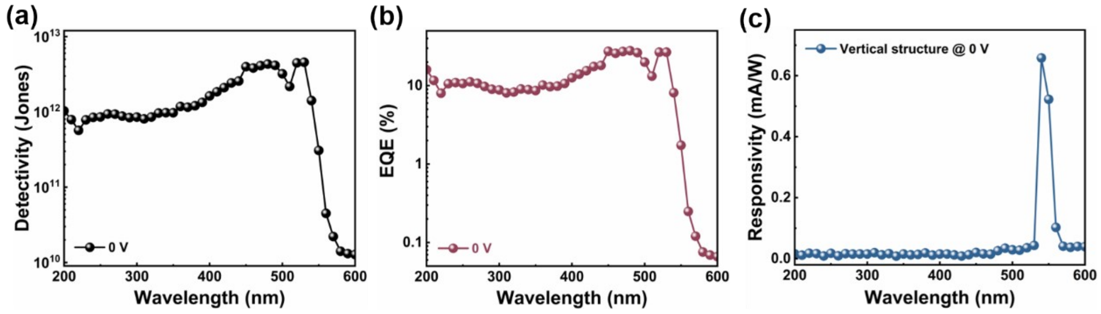

Fig. S6 (a) The detectivity spectrum of Pt-Ag photodetector at 0 V. (b) The EQE spectrum of the Pt-Ag photodetector at 0 V. (c) The photoresponse curve of the Pt-Ag photodetector with vertical structure at 0 V.

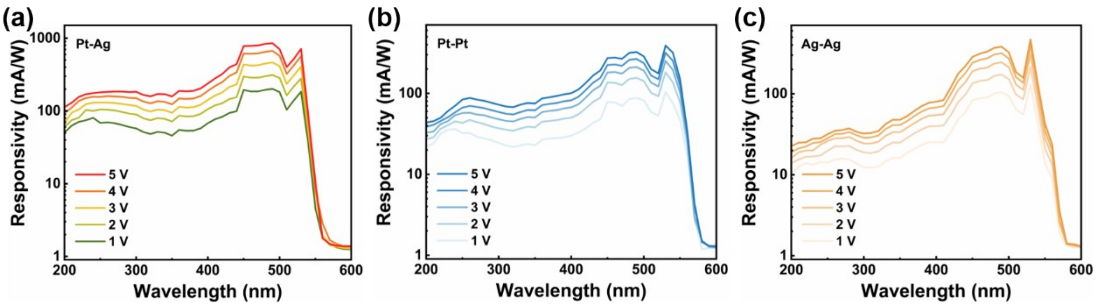

Fig. S7 (a-c) The responsivity spectra of the Pt-Ag, Pt-Pt and Ag-Ag photodetectors at different bias voltage, respectively.

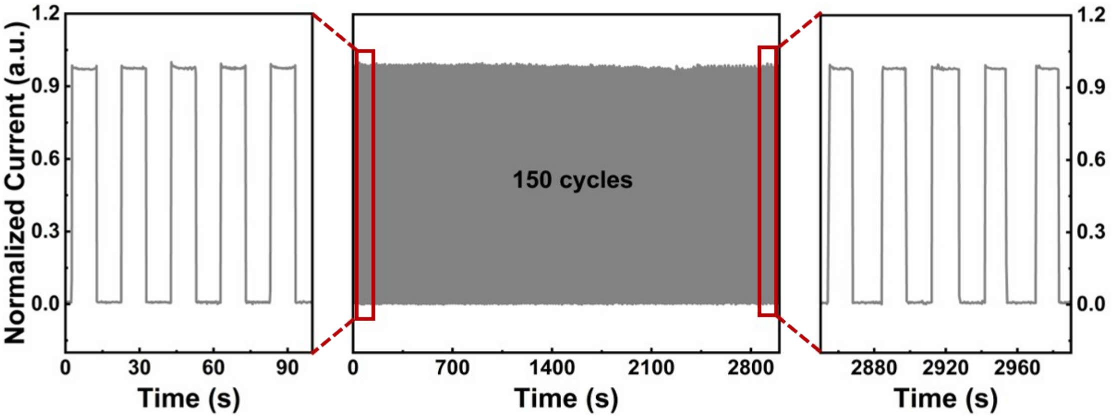

Fig. S8 Long-term *I*-*t* curves of the Pt-Ag photodetector at 0 V.

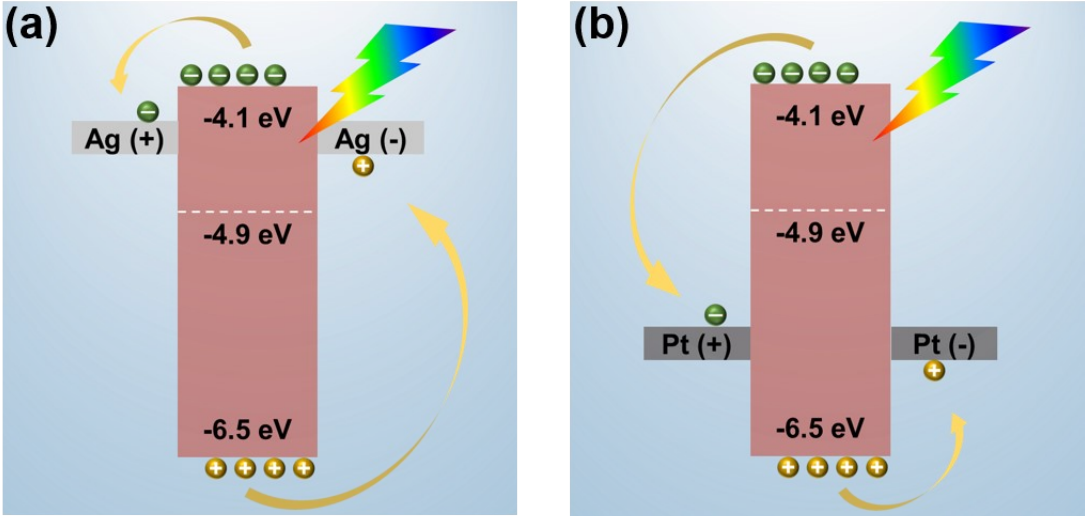

Fig. S9 Energy band diagrams and charge carrier transfer of the devices with (a) Ag-Ag and (b) Pt-Pt symmetric electrodes upon light illumination.

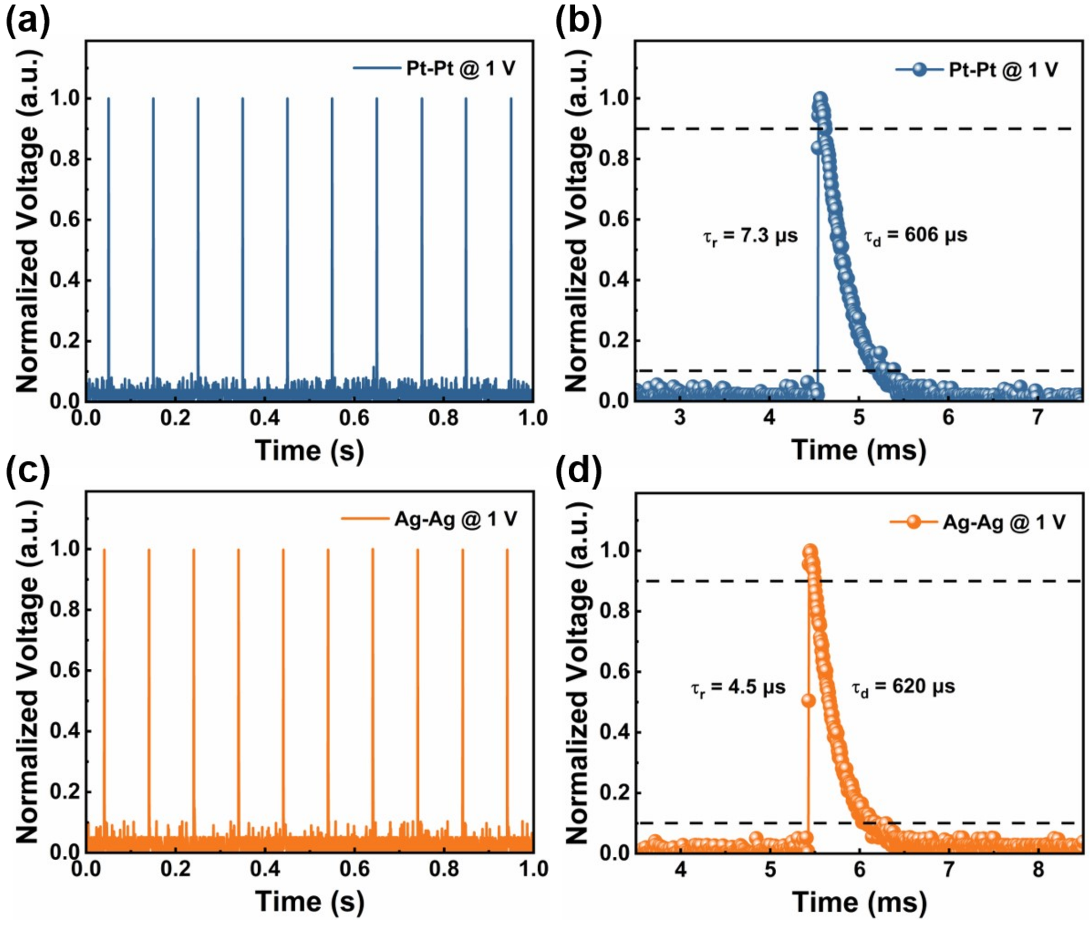

Fig. S10 The time-resolved response at 1 V under the excitation of 355 nm pulsed laser: (a) and (b) the Pt-Pt detector. (c) and (d) the Ag-Ag detector.

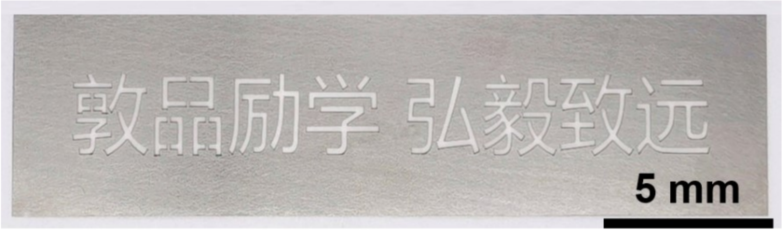

Fig. S11 The image for the object in a steel foil.
